# Mating Increases Neuronal Tyrosine Hydroxylase Expression and Selectively Gates Transmission of Male Chemosensory Information in Female Mice

**DOI:** 10.1371/journal.pone.0069943

**Published:** 2013-07-25

**Authors:** Gillian A. Matthews, Ronak Patel, Alison Walsh, Owain Davies, Joana Martínez-Ricós, Peter A. Brennan

**Affiliations:** 1 School of Physiology and Pharmacology, University of Bristol, Bristol, United Kingdom; 2 Department of Human Anatomy, Universitat de València, València, Spain; Duke University, United States of America

## Abstract

Exposure to chemosensory signals from unfamiliar males can terminate pregnancy in recently mated female mice. The number of tyrosine hydroxylase-positive neurons in the main olfactory bulb has been found to increase following mating and has been implicated in preventing male-induced pregnancy block during the post-implantation period. In contrast, pre-implantation pregnancy block is mediated by the vomeronasal system, and is thought to be prevented by selective inhibition of the mate’s pregnancy blocking chemosignals, at the level of the accessory olfactory bulb. The objectives of this study were firstly to identify the level of the vomeronasal pathway at which selective inhibition of the mate’s pregnancy blocking chemosignals occurs. Secondly, to determine whether a post-mating increase in tyrosine hydroxylase-positive neurons is observed in the vomeronasal system, which could play a role in preventing pre-implantation pregnancy block. Immunohistochemical staining revealed that mating induced an increase in tyrosine-hydroxylase positive neurons in the arcuate hypothalamus of BALB/c females, and suppressed c-Fos expression in these neurons in response to mating male chemosignals. This selective suppression of c-Fos response to mating male chemosignals was not apparent at earlier levels of the pregnancy-blocking neural pathway in the accessory olfactory bulb or corticomedial amygdala. Immunohistochemical staining revealed an increase in the number of tyrosine hydroxylase-positive neurons in the accessory olfactory bulb of BALB/c female mice following mating. However, increased dopamine-mediated inhibition in the accessory olfactory bulb is unlikely to account for the prevention of pregnancy block to the mating male, as tyrosine hydroxylase expression did not increase in females of the C57BL/6 strain, which show normal mate recognition. These findings reveal an association of mating with increased dopaminergic modulation in the pregnancy block pathway and support the hypothesis that mate recognition prevents pregnancy block by suppressing the activation of arcuate dopamine release.

## Introduction

Mice learn to recognise their mate’s chemosignals during a sensitive period for memory formation contingent on mating [1]. This recognition of the mating male is vital for reproductive success, as it prevents pregnancy failure (the Bruce effect), which is elicited by exposure to unfamiliar male chemosignals during the pre-implantation period, up to 4 days following mating [2]. The neural changes underlying mate recognition are thought to occur in the accessory olfactory bulb (AOB), which projects, via the corticomedial amygdala, to the arcuate hypothalamus [3]. Activation of this pregnancy-blocking pathway by unfamiliar male chemosignals results in the release of dopamine from the arcuate hypothalamus, which inhibits prolactin release from the pituitary, thereby removing luteotrophic support and resulting in implantation failure [4,5]. Mate recognition during the pre-implantation period can be explained simply by the selective inhibition of the response to mating male chemosignals, at the level of the AOB, suppressing their effectiveness in eliciting dopamine release from the arcuate hypothalamus [1]. Consistent with this hypothesis, both electrophysiological recordings and analysis of c-Fos expression have revealed a selective suppression of responses in the medial amygdala following exposure to mating male compared to unfamiliar male chemosignals [6,7]. The first objective of this study was to use immunohistochemistry for c-Fos to determine whether a differential response could also be observed at other levels of the pregnancy blocking pathway including the AOB input and the arcuate neuroendocrine output. Our study found a selective suppression of c-Fos response to the mating male pheromones in the tyrosine hydroxylase (TH)-positive dopaminergic neurons in the arcuate hypothalamus, but not at other levels of the pregnancy blocking pathway. Unexpectedly, we found that mating was also associated with an increase in the number of TH-positive neurons in the arcuate nucleus.

A recent study found an increase in the number of tyrosine hydroxylase (TH)-positive dopaminergic periglomerular cells in the main olfactory bulb (MOB), during the post-implantation period, 4-8 days following mating [8]. Furthermore, this increased dopaminergic inhibition of social odour input to the MOB, prevented male odours from blocking pregnancy during the post-implantation period [8]. Serguera et al. (2008) failed to find any TH-positive neurons in the AOB of the C57BL6 mice in their study. However, our initial experiments revealed a small number of TH-positive neurons in the AOB of BALB/c mice, consistent with previous reports [9,10]. This raised the possibility that mating might increase the number of TH-positive neurons in the AOB, and that enhanced dopaminergic modulation of vomeronasal input to the AOB might play a role in selectively preventing pre-implantation pregnancy block to mating male chemosignals. We therefore extended the study in a separate experiment to determine whether the number of TH-positive neurons increased in the AOB following mating, similar to the post-mating increases observed in the MOB and arcuate hypothalamus. We observed an increase in TH-positive neurons in the AOB following mating and found that a strain difference could at least partly explain Serguera et al.’s previous failure to observe TH expression in the AOB [8]. We conclude that post-mating increase in dopaminergic transmission in the AOB has the potential to modulate vomeronasal sensory input. However, there are a number of reasons why this is unlikely be the mechanism underlying mate recognition in the Bruce Effect.

## Materials and Methods

Adult BALB/c and C57BL/6 female (20–25 g) and male (2-6 months) mice (Harlan, UK) were housed on pine chip bedding under a 12:12 reversed light cycle with lights off at 08:00, with free access to food and water.

### Ethics statement

All procedures were performed in accordance with the UK Animals (Scientific Procedures) Act 1986 (Project Licence Number 30/2519), and approved by the University of Bristol Ethical Review Committee.

### Effect of mating on c-Fos expression in the pregnancy block pathway

Proestrus/oestrus BALB/c females were randomly assigned, in blocks, to four groups (n=8 per group), by drawing lots. Half of the females in each of the Control and the Exposed groups were placed in a cage containing BALB/c male bedding and the other half of the females were placed in a cage containing C57BL/6 male bedding. Such exposure without mating is insufficient for memory formation and subsequent male recognition [1]. Half of the females in both the Mated Familiar and the Mated Unfamiliar groups were mated with a BALB/c male in his home cage, whilst the other half of the females were mated with a C57BL/6 male in his home cage. This counterbalancing of the strain of male exposure/mating within each group controlled for the potentially confounding effect of different c-Fos responses to chemosignals from different strains of mating and unfamiliar males. Mating was confirmed by detection of a vaginal plug. Females were removed from the mating male and/or male chemosignals after 5 hours and housed in pairs, in clean cages, with similarly treated females. Two days following mating/exposure, at a time when pregnancy block is known to occur [2], females received a 90-minute bedding exposure, before being killed for immunohistochemistry. Control females were exposed to clean bedding. Exposed females were re-exposed to male bedding from the same strain to which they had been pre-exposed. Mated Familiar females were re-exposed to bedding from the same strain as the mating male. Mated Unfamiliar females that had mated with a C57BL/6 male were exposed to bedding from a BALB/c male, and *vice versa*.

### Effect of mating on TH expression in the AOB

Females in proestrus/oestrus were pseudorandomly allocated to one of six groups. Females in mated groups (3 BALB/c and 3 C57/BL6 per group) were introduced to a BALB/c male in his home cage and mating was confirmed by the presence of a vaginal plug. Five hours later, females were transferred to a clean cage containing 2-3 other mated females. Females in groups M1, M2, M4 and M8/9 were killed at days 1, 2, 4 and 8 or 9 following mating, respectively. Females in the baseline control group, T0 (4 BALB/c, 2 C57BL/6) were killed without mating or male exposure. To determine whether the changes observed were dependent on mating, females in group E4 (2 BALB/c, 4 C57BL/6) were exposed to BALB/c male bedding for 5 hours without mating and killed 4 days later.

### Immunohistochemistry

Females were terminally anaesthetised with an overdose of ketamine-xylazine anaesthetic (0.3 and 0.03 mg/kg) and perfused transcardially with 5ml of phosphate buffered saline followed by 20ml of freshly depolymerised 4% paraformaldehyde in phosphate buffered saline (pH 7.3). Brains were removed and post-fixed for 4 hours before being transferred to 30% sucrose in phosphate buffered saline, and left overnight at 4^°^C. 40 µM frozen sagittal and coronal sections were cut through the AOB and amygdala/hypothalamus, respectively. Endogenous peroxidase activity was blocked by incubating for 10 minutes in 8.8% methanol, 3.5% hydrogen peroxide in phosphate buffered saline with 0.3% Triton X-100. Standard immunohistochemistry was performed, at room temperature, on free-floating sections [11].

For the c-Fos experiment, AOB and amygdala/hypothalamus sections were incubated for one hour in 1:1000 dilution of anti-c-Fos rabbit polyclonal antibody (sc-52, Santa Cruz Biotechnology, California, http://antibodyregistry.org/AB_2106782), followed by a 30-minute incubation in Vector ImmPRESS® and visualised using SG peroxidase substrate (Vector laboratories). Alternate coronal sections were incubated for a further hour in 1:2000 dilution of anti-tyrosine hydroxylase rabbit polyclonal antiserum (sc-14007, Santa Cruz Biotechnology), followed by a 30-minute incubation in Vector ImmPRESS® and visualised using DAB peroxidase substrate (Vector laboratories).

For the mating-TH experiment, AOB sections were incubated for one hour in a 1:400 dilution of the same mouse monoclonal anti-tyrosine hydroxylase antibody (MAB318, Millipore, Massachusetts, http://antibodyregistry.org/AB_94738) used by Serguera et al. [8]. This was followed by a 30-minute incubation in 1:1000 dilution of biotinylated rat anti-mouse IgG1 heavy chain (MCA336B, AbD Serotec, Kidlington, UK) second antibody, and visualized using a Vectorstain Elite ABC kit and Vector SG peroxidase substrate (Vector Laboratories, California). Omission of primary antibody resulted in no staining. Sections were mounted on gelatin-coated slides and counterstained using neutral red to enable delineation of the borders of the AOB. A similar protocol was performed on sagittal AOB sections from an additional animal using a rabbit polyclonal anti-dopamine beta hydroxylase antibody (ab43868, Abcam, Cambridge, UK, http://antibodyregistry.org/AB_731852), 1:1000 overnight incubation. Staining was visualized using a Vectastain ABC kit and SG peroxidase substrate (Vector Laboratories, California).

### Data collection and statistical analysis

All slides were coded and analysis was done blind to animal treatment. Statistical analyses were performed using SPSS 18 (IBM, New York). Arcuate TH-positive neurons, with and without a c-Fos positive nucleus, were identified in three alternate, double-labelled coronal sections through the central arcuate hypothalamus, under X400 magnification (Olympus BHS microscope). Cells were marked on a captured image using a cell counting script in ImageJ [12]. Differences in the percentage of double-labelled neurons among groups were assessed using two-factor (group x strain) and single-factor (group) ANOVA with Tukey *post-hoc* multiple comparisons, following log transformation to meet parametric assumptions. Data from Control and Exposed groups and from Mated Familiar and the Mated Unfamiliar groups were combined to analyse the effect of mating on the number of TH-positive neurons counted in the arcuate hypothalamus.

ImageJ was also used to count c-Fos-positive nuclei in the amygdala of 4 alternate, single-labelled coronal sections. Greyscale images were filtered using rolling ball background subtraction and segmented using k-means clustering into 20 greyscale bands. The greyscale band containing the c-Fos-positive nuclei was selected, a circular area of constant size was positioned centrally in the relevant amygdala sub-region [13] and the number of stained nuclei counted. The number of c-Fos-positive nuclei were similarly counted in 3 alternate sagittal sections through the central region of the AOB, using 8 greyscale bands, using a freehand selection of anterior and posterior sub-regions, for both mitral cell and glomerular layers separately. Counts of c-Fos-positive nuclei for both amygdala and AOB were converted to number per mm^2^ by dividing by the area of the counting selection. Data on stained nuclei per mm^2^ for both AOB and amygdala met parametric assumptions and were analysed by either two-factor (group x strain) or single-factor (group) ANOVA for each amygdala sub-region, or for the AOB by three-factor (group x sub-region x strain) or two-factor (group x sub-region) mixed model ANOVA, with anterior/posterior as a repeated measure. Tukey *post-hoc* multiple comparisons or Dunnett’s test were used, where appropriate.

For the TH-mating experiment, the anterior and posterior borders of the AOB were easily identified in the neutral red, counterstained sections. However, the medial and lateral borders were more difficult to determine in sagittal sections, especially on the medial side, where it merged with the MOB. Therefore only the 16 consecutive sections through the central region of the AOB in which the glomerular, mitral cell and granule cell layers could all be defined clearly were included in the analysis. Notably we found a high density of TH-positive neurons encircling the vomeronasal nerve in more medial sections, which were not included in our analysis. Furthermore we took a conservative approach in counting only TH-positive neuronal somata that were clearly within the anterior–posterior boundaries of the AOB. This criterion excluded neurons located on the anterior border of the AOB even if their dendritic tree extended into the AOB. These conservative criteria are thus likely to have underestimated the actual number of TH-positive neurons that could influence AOB neural activity. The number of TH-positive somata were counted by eye under X400 magnification separately for anterior and posterior sub-regions, and the totals from right and left AOBs were averaged for subsequent analyses. Data were analyzed using a mixed-model three-factor repeated measures ANOVA with sub-region as a within-subjects factor and with group and strain as between-subject factors. Splitting the groups of 6 into the two strains enabled us to analyse whether a strain difference affected the differences in number of TH-positive neurons across groups. The resultant group sizes were too small for Tukey post-hoc multiple comparisons. Therefore more detailed analysis proceeded by pooling data into groups of mated (BALB/c n=12, C57BL/6 n=12) and non-mated females (BALB/c n=6, C57BL/6 n=6) to analyse the effect sizes of mating, strain and anterior–posterior sub-region on number of TH-positive neurons, using unpaired t-tests, or Mann–Whitney U tests where parametric assumptions were not met.

## Results

Two-factor ANOVAs revealed that there was no significant effect of the strain of male to which the females were initially mated/exposed on the number of TH-positive neurons (strain x group, F_3,24_ = 1.50, p = 0.238) or the percentage of TH-positive neurons with c-Fos co-localisation (strain x group, F_3,24_ = 0.19, p = 0.899) in the arcuate. There was also no significant effect of the strain of male to which the females were initially mated/exposed on c-Fos expression in amygdala sub-regions: MePD (strain x group, F_3,24_ = 0.53, p = 0.665); MePV (strain x group, F_3,24_ = 0.49, p = 0.692); PMCo (strain x group, F_3,24_ = 0.03, p = 0.992). The factor of strain was thus disregarded in further analysis of these data.

### Mating increased the number of arcuate dopaminergic neurons, but decreased their response to mating male chemosignals

**Figure 1:**
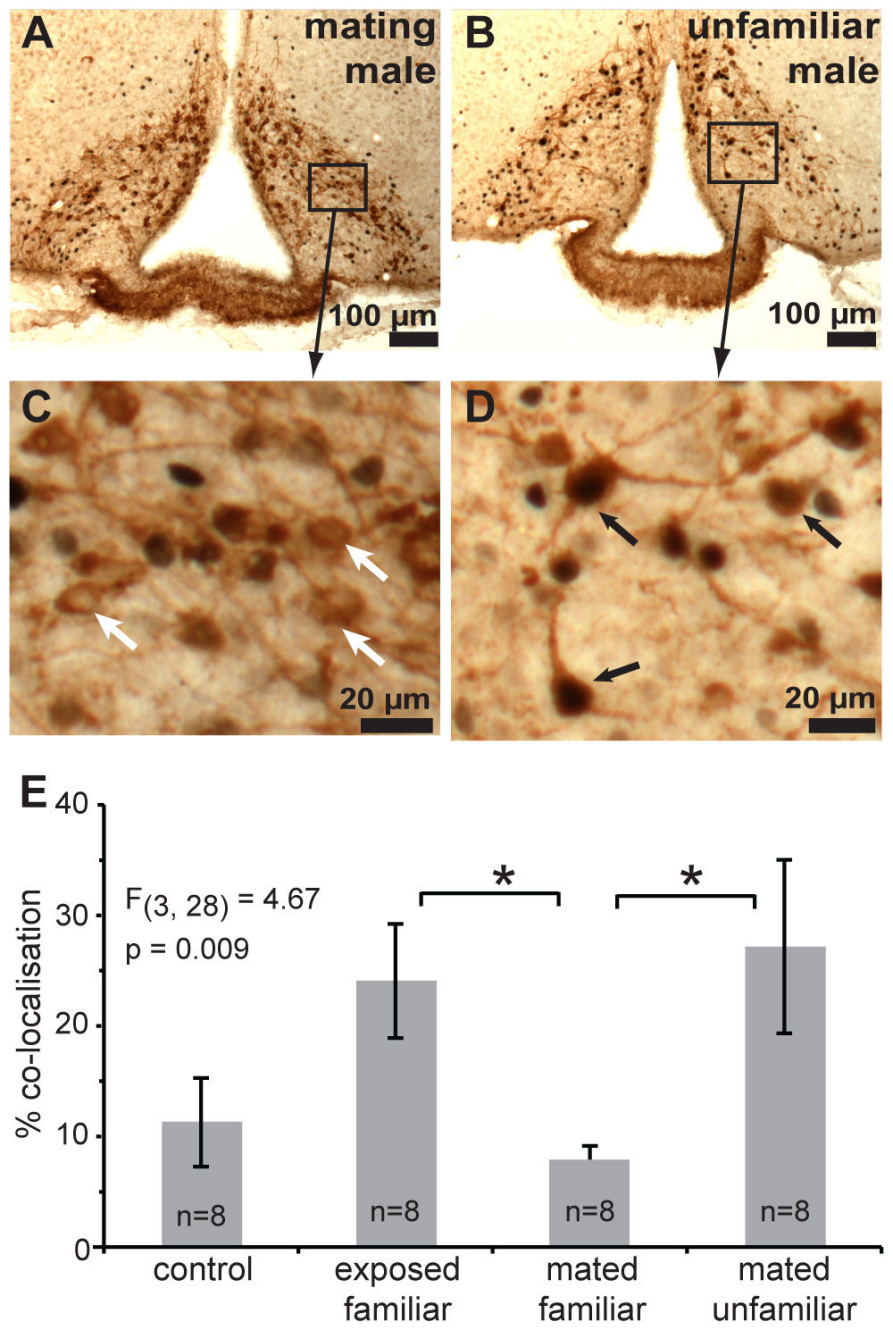
Mating suppresses the effectiveness of mating male’s chemosignals in activating arcuate dopaminergic neurons. Significantly lower co-localisation of c-Fos (black nuclei) was found in dopaminergic arcuate neurons (brown cytoplasm) of mated females exposed to familiar (mating) male bedding (A, C), than to bedding from an unfamiliar male (B, D). Black and white arrows indicate tyrosine hydoxylase-positive neurons with and without c-Fos co-localisation respectively. E) Mean (± standard error) percentage of arcuate dopaminergic neurons with c-Fos co-localisation was significantly lower in mated females exposed to the mating male compared to an unfamiliar male, or unmated females re-exposed to male bedding (*p < 0.05, Tukey multiple comparisons).

The mean number of arcuate TH-positive neurons that were counted in the three coronal sections differed significantly among the four groups *F*_3,28_ = 4.28, *p* = 0.0133. When groups were combined, the mean number of TH-positive arcuate neurons counted in mated females (320.1 ± 13.9), two days following mating, was significantly higher than in the females that received an equal amount of prior exposure to male bedding without mating (255.9 ± 12.8, effect size r = 0.53, *t*_30_ = 3.4, *p* = 0.002). The effectiveness of male bedding exposure in activating these neurons was assessed by calculating the percentage of TH-positive neurons that contained a c-Fos-positive nucleus, which differed significantly among the four bedding exposure groups *F*_3,31_ = 4.69, *p* = 0.009 (Figure 1). Exposure to mating male bedding was ineffective in activating dopaminergic arcuate neurons, as *post-hoc* multiple comparisons revealed that the mean percentage c-Fos-TH co-localisation was significantly lower in females exposed to mating male bedding compared to mated females exposed to unfamiliar male bedding and compared to unmated females re-exposed to male bedding (Figure 1).

### No differential c-Fos expression to mating male chemosignals in the AOB and amygdala

**Figure 2:**
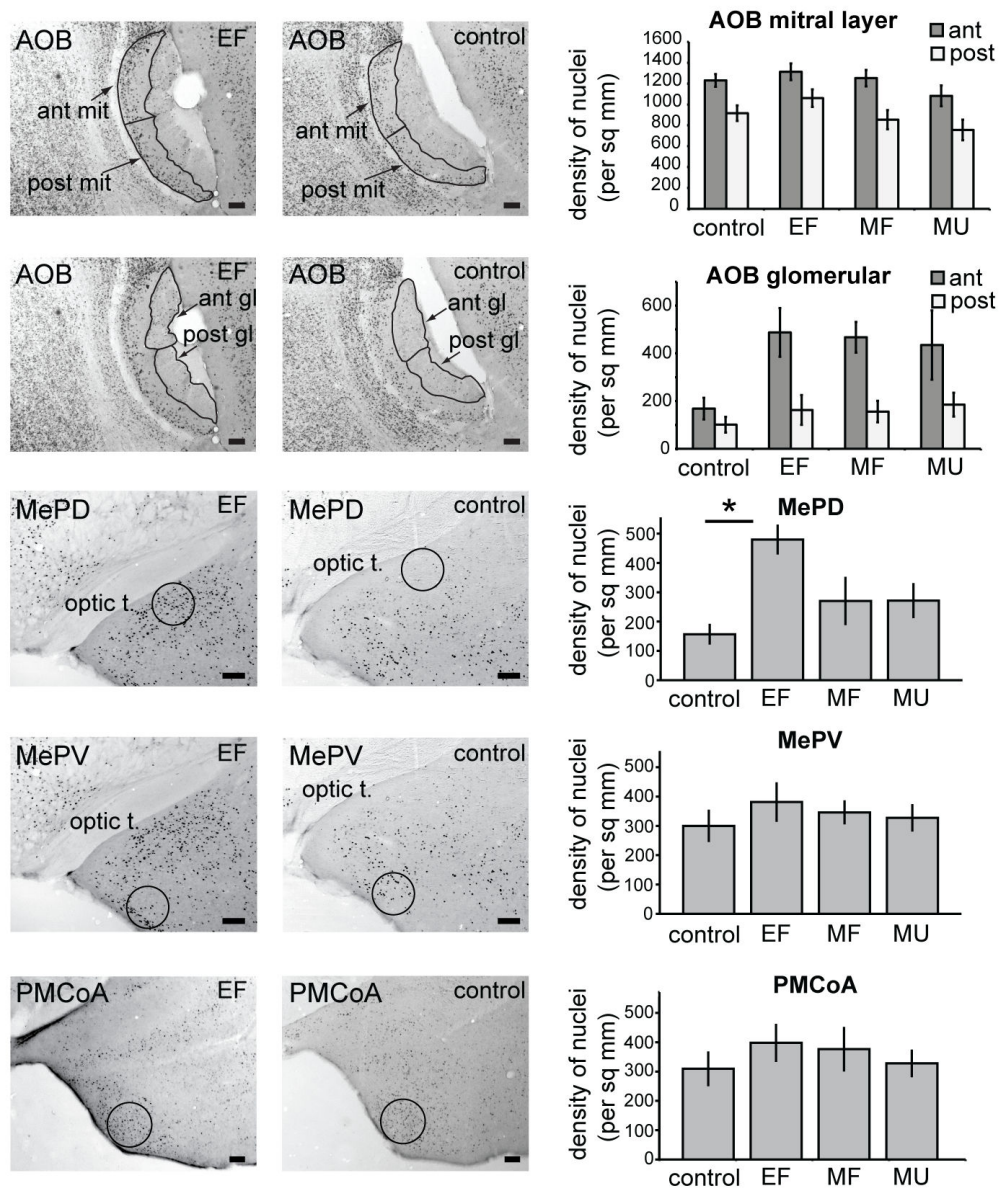
Lack of a differential c-Fos response to male bedding in the AOB or amygdala. Representative examples of c-Fos immunohistochemistry and the location of counting areas for non-mated females re-exposed to male bedding (EF, left) and for non-mated females exposed to clean bedding (control, centre). Right panel shows mean cell counts per mm^2^ (± standard error) of c-Fos-positive nuclei in AOB and amygdala of control and EF groups, as well as groups of mated females that were re-exposed to mating male bedding (MF) or exposed to bedding from an unfamiliar strain of male (MU) two days following mating (*p < 0.05, Tukey multiple comparisons). AOB, accessory olfactory bulb (ant, anterior sub-region; post, posterior sub-region); mit, mitral cell layer; gl, glomerular layer); MePD, posterodorsal medial amygdala; MePV, posteroventral medial amygdala; PMCoA, posteromedial cortical amygdala; optic t, optic tract.

In contrast to the selective suppression of the response to mating male chemosignals we observed in the arcuate, we found no evidence for selective c-Fos responses to mating male vs unfamiliar male bedding at either the AOB or amygdala levels of the pregnancy-blocking pathway (Figure 2). There was a significant difference among groups in the mean number of c-Fos nuclei per mm^2^ in the posterodorsal medial amygdala (MePD, *F*_3,28_ = 5.11, *p* = 0.006), with male bedding exposure causing a significant increase in mean count of c-Fos-positive nuclei in non-mated females compared to the control group (Figure 2). However, there was no differential response to mating male bedding compared to unfamiliar male bedding in the MePD of mated females. There was no significant effect of male exposure on c-Fos expression in the posteroventral medial amygdala or the posteromedial cortical amygdala of mated or unmated females. Furthermore, there was also no differential response to the mating male compared to unfamiliar male bedding in the AOB (Figure 2).

Repeated measures ANOVA revealed a significantly higher density of c-Fos positive nuclei in the anterior compared to posterior sub-region of the AOB, for both glomerular layer (*F*_1,27_ = 31.45, *p* < 0.001) and mitral cell layer (*F*_1,27_ = 53.94, *p* < 0.001), but this appeared to be an intrinsic difference in c-Fos expression between the sub-regions, as it was not affected by exposure to male chemosignals. A significant interaction was found among strain, group and sub-region for the number of c-Fos positive nuclei in the AOB mitral cell layer (*F*_3,23_ = 4.87, p = 0.009). This was due to the tendency for a higher number of c-Fos nuclei in the anterior sub-region of the exposed group of BALB/c females compared to the exposed group of C57BL/6 females and lower numbers in the control and mate familiar group of BALB/c versus C57BL/6 females (Figure S1). Further analysis using single-factor ANOVA revealed a significant effect of group on stained mitral nuclei in the anterior AOB of females that were initially mated/exposed to BALB/c male chemosignals (*F*_3,12_ = 3.70, p = 0.043, with large effect size r = 0.64), with a significantly higher number of nuclei in exposed compared to control group (p = 0.017, Dunnett t test for > control). There was also a significant interaction between strain and sub-region for the number of c-Fos positive nuclei in the AOB glomerular layer *F*_1,23_ = 4.56, p = 0.044, medium effect size r = 0.40. This appears to be due largely to a higher number of c-Fos positive nuclei in the anterior, but not posterior, sub-region of females that were initially mated/exposed to C57BL/6 male chemosignals (Figure S1).

### Increase in TH-expressing AOB neurons following mating

Serguera et al.’s [8] findings raised the interesting possibility that increased dopaminergic transmission in the AOB following mating could selectively inhibit the transmission of chemosensory information from the mating male. Our initial experiments, using the same rabbit polyclonal anti-TH antiserum (sc-14007) used to identify arcuate dopaminergic neurons in the first experiment, revealed TH-positive neurons in the AOB of BALB/c female mice (not shown). We therefore sought to determine whether the number of these TH-positive neurons was increased following mating, and the basis for the discrepancy with Serguera et al.’s findings (Serguera et al., 2008). To eliminate the possibility that Serguera et al’s failure to find TH-positive neurons in the AOB could be due to differences in the primary antibody used, we used the same mouse monoclonal anti-tyrosine hydroxylase antibody (MAB318) used by Serguera et al. (2008). We also used the same basic experimental design except for including both BALB/c and C57BL/6 females in each group to additionally test whether a strain difference could explain the the discrepancy in the findings.

**Figure 3:**
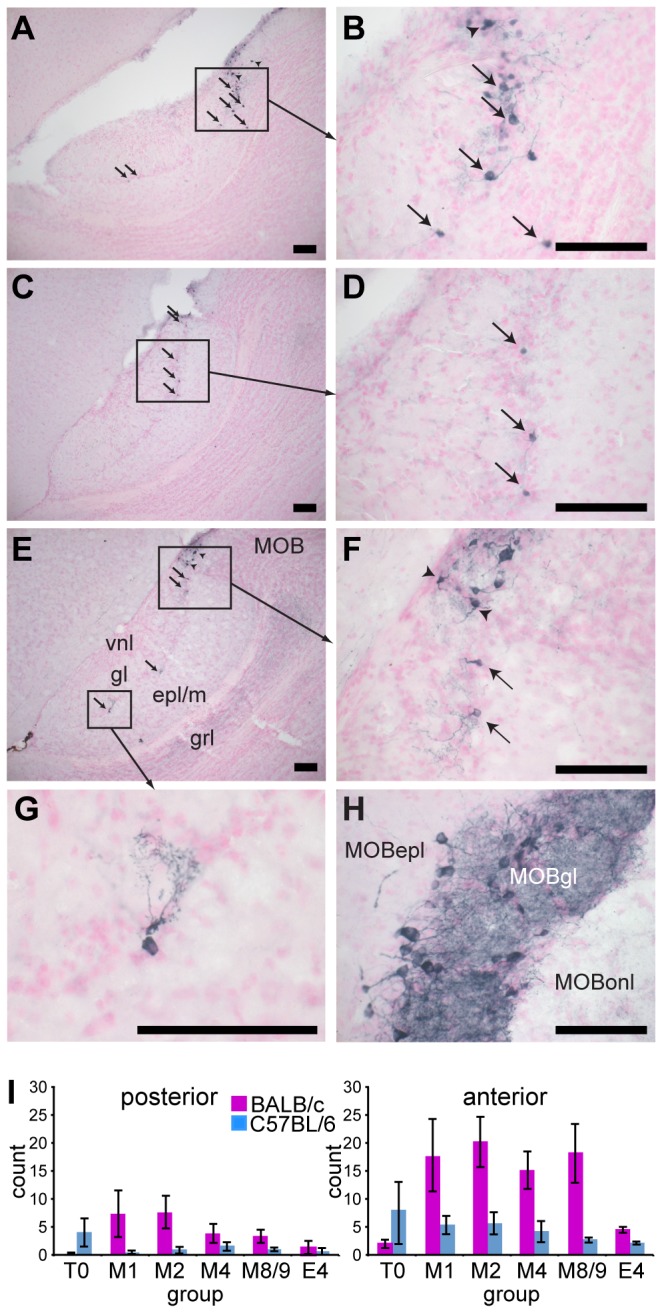
Mating increased the number of TH-positive neurons in the AOB of BALB/c mice. Sagittal sections through the AOB of: BALB/c female 2 days following mating (A, B); BALB/c female 9 days following mating (C, D); C57BL/6 female exposed to male bedding (day 0) without mating (E-G). Arrows indicate TH-positive neurons counted within the AOB. Arrowheads indicate TH-positive neurons at the boundary of the AOB that were not counted, despite extending dendrites into the AOB. H) TH-positive neurons were abundant in the main olfactory bulb (MOB). I) Mean (± standard error) number of TH-positive neurons per AOB was increased in mated BALB/c (M1-8/9), but not C57BL/6, females, compared to females without male exposure (T0) and 4 days following exposure to male chemosignals without mating (E4). Scale bars indicate 0.1 mm. AOB structures: vnl, vomeronasal nerve layer; gl, glomerular layer; epl/m, external plexiform/mitral cell layer; grl, granule cell layer. MOB structures: MOBepl, external plexiform layer; MOBgl, glomerular layer; MOBonl, olfactory nerve layer.

We observed TH-positive neurons in the AOB of both BALB/c and C57BL/6 female mice (Figure 3). Although there were far fewer TH-positive neurons in the AOB than found in the MOB, they had a similar glomerular layer location, with dendrites innervating localised glomerular regions (Figure 3). ANOVA revealed a significant sub-region x strain x mating interaction, *F*_5,24_ = 3.03, *p* = 0.029, suggesting a significant difference between the strains in the effect of mating on the number of TH-positive neurons in the AOB. As can be seen in Figure 3 this is predominantly due to an increase in the number of TH-positive neurons following mating in the anterior AOB of mated BALB/c females. More detailed analysis, revealed a significantly higher number of TH-positive neurons in the anterior AOB of the 24 mated females compared to the anterior AOB of the 12 non-mated females (effect size r = 0.53, U_24,12_ = 237.5, *p* = 0.001, mean difference = 7.8 cells). There was also a lower, but still significant increase in the number of TH-positive neurons in the posterior AOB of mated compared to non-mated females (effect size r = 0.39, U_24,12_ = 212.5, *p* = 0.020, mean difference = 2.1 cells). There was a significantly higher number of TH-positive neurons in the AOB of mated BALB/c (mean of 23.4 ± 3.3) than mated C57BL/6 females (mean difference of 5.4 ± 0.9, r = 0.78, U_12,12_ = 6.00, *p* < 0.001). However, there was no significant difference between the number of TH-positive neurons between mated and non-mated C57BL/6 females. There were significantly higher mean numbers of TH-positive neurons in the anterior compared to posterior AOB in mated females of both BALB/c (mean difference 12.3 ± 1.4, r = 0.88, *T*_12_ = 0, *p* < 0.001) and C57BL/6 strains (mean difference 3.2 ± 0.7, r = 0.88, *T*_12_ = 0, *p* < 0.001).

No staining of neuronal cell bodies in either the AOB or MOB was observed with the anti-dopamine beta hydroxylase antibody (not shown). Coronal sections through the pons were used as a positive control and revealed strong staining for dopamine beta hydroxylase in noradrenergic neurons in the locus caeruleus (not shown). These results suggest that TH-positive neurons in the AOB are likely to be dopaminergic rather than noradrenergic or adrenergic, similar to the dopaminergic periglomerular cells in the MOB.

## Discussion

This study is the first to report an influence of mating on TH expression in the mouse arcuate hypothalamus and AOB of female mice, suggesting the potential for mating-dependent modulation of vomeronasal function. TH expression is known to be hormonally regulated, with TH expression in the rat arcuate being inhibited by oestrogens and enhanced by progesterone and prolactin [14,15]. In mice the vaginocervical stimulation at mating acts at the hypothalamus to induce twice daily peaks of prolactin release from the anterior pituitary [16]. These have a luteotrophic effect to maintain ovarian progesterone production. These increased levels of prolactin and progesterone are likely to mediate the post-mating increases in TH in the AOB and arcuate hypothalamus observed in the BALB/c mice in this study.

### Gating of male chemosignals in the vomeronasal pathway

TH-c-Fos co-localisation in arcuate neurons is associated with increased dopamine release, and is inversely correlated with serum prolactin peaks [17]. Hence the increased degree of arcuate TH-c-Fos co-localisation in response to unfamiliar male chemosignals is consistent with a reduction in serum prolactin levels, removing luteotropic support and leading to pregnancy block. The increase in TH-positive neurons in the arcuate hypothalamus of mated female mice has the potential to enhance arcuate dopamine release, increasing a mated female’s susceptibility to pregnancy block [3,5,17] This highlights the importance for reproductive success of a robust mechanism for suppressing activation of this dopaminergic output during exposure to chemosignals from the mating male. Our finding that exposure to mating male’s chemosignals was ineffective in increasing TH-c-Fos co-localisation in arcuate neurons in comparison with unfamiliar male chemosignals suggests that transmission of the mate’s chemosignals is indeed selectively suppressed, preventing activation of the arcuate pregnancy-blocking output [1]. Our findings contrast with previous studies that have failed to observe appreciable arcuate c-Fos expression in response to male chemosignals [7,8]. However, these studies also reported less than half the number of TH-positive neurons per arcuate section than we observed, suggesting that their failure to observe c-Fos expression could be due to a lower overall sensitivity of their immunohistochemical procedures.

Both pre-implantation pregnancy block and mate recognition in mice are mediated by the vomeronasal system [18,19]. However, although we found a significant c-Fos response to male bedding in the MePD of non-mated females, there was no evidence for a differential response to mating versus unfamiliar male bedding in the MePD, posteroventral medial amygdala or posterocortical medial amygdala of mated females, all of which receive a direct projection from the AOB. These differ from Halem et al.’s [7] findings of a suppression of the c-Fos response to mating male bedding in targets of AOB projections, including the MePD, which is thought to be involved in a neural pathway mediating reproductive chemosensory stimuli in mice [20,21]. It also contrasts with our previous findings of lower spike rates in medial amygdala neurons in response to mating versus unfamiliar male urine [6]. The reason for these discrepancies is unclear, as although the counting regions in this study were generally more posterior than the electrophysiological recording sites, they were similar to those used by Halem et al [7]. Different inbred strains of male have been reported to differ in their pregnancy blocking effectiveness [22]. However, the BALB/c and C57BL/6 strains of male used in this study are both effective in blocking pregnancy [23] and are the same strains used by Halem et al [7]. Furthermore, we found no effect of strain of the mating/exposure male chemosignals on c-Fos levels in the amygdala or arcuate. Our ability to detect a differential response in the amygdala may have been limited by the high basal level of c-Fos positive nuclei we observed in control females. These basal levels are noticeably higher than found in other studies [7]. But they are consistent with the high control levels that we observed in the AOB of this study and that we have previously observed using a different c-Fos primary antibody and different animal housing conditions [11]. The relatively high sensitivity of our immunohistochemical staining procedure, which enabled the detection of differential c-Fos responses in the arcuate, may have constrained our ability to detect differential c-Fos responses in the amygdala.

We also found no overall evidence for a differential response to mating male and unfamiliar male chemosignals in the AOB of mated females, although in this case there was a significant influence of the strain of male chemosignal pre-exposure. As the initial mating/exposure to male chemosignals occurs two days before the test exposure, it is difficult to interpret the effects of strain on number of c-Fos positive nuclei in the mitral and glomerular layers of the AOB. It is conceivable that there could be a strain dependent priming or habituation effect of the first mating/exposure that influences their subsequent response to chemosensory exposure. But, this requires confirmation in future experiments. The significantly higher density of c-Fos positive nuclei in the anterior sub-region of both the glomerular and mitral cell layers of the AOB in response to soiled male bedding. is likely to be an intrinsic property of the AOB and is consistent with previous observations [7].

### Mating enhances TH expression in the AOB

TH is the rate-limiting enzyme in dopamine synthesis and the lack of dopamine beta hydroxylase immunostaining in the AOB suggests that the TH-positive neurons observed in the AOB were likely to have been dopaminergic, rather than noradrenergic. A sub-population of olfactory bulb interneurons differentiate into dopaminergic neurons and express TH mRNA, but not protein, before they integrate into olfactory bulb circuits, during their migration from the rostral migratory stream [24]. Therefore the post-mating increase in TH-expressing neurons in both the AOB and MOB is most likely due to an increase in TH protein content in neurons with pre-existing dopaminergic differentiation, rather than a recruitment of non-dopaminergic neurons.

What role, if any, might these TH-positive neurons have on vomeronasal processing in the AOB? The compact dendritic trees of TH-positive neurons appear to project to localised glomerular targets, mainly in the anterior AOB. The sparseness of TH-positive periglomerular neurons suggests that they may play a role in selectively modulating input from specific vomeronasal receptor types, potentially via pre-synaptic inhibition of input from the vomeronasal nerve by a similar D_2_-receptor mechanism to that found in the MOB [25,26]. Afferent activity increases TH expression in MOB periglomerular cells, and hence D_2_-mediated dopaminergic inhibition, which acts to stabilise the overall level of sensory input to the MOB [25,26]. However, in contrast to the more general odour representation function of the MOB [27], the AOB senses specialised chemosignals, which are likely to be hardwired to specific responses [28]. Therefore, unlike the distributed and overlapping patterns of glomerular activation elicited by odour stimuli in the MOB [29], AOB glomeruli are likely to respond more selectively to specific vomeronasal stimuli [30]. The low number of TH-positive neurons in the AOB might therefore be a consequence of low levels of vomeronasal stimulation in laboratory housing conditions [31], reflecting low levels of TH protein expression in the AOB, despite substantial levels of transcription from the TH promotor [24]. This sparseness of the TH-positive neurons in the AOB [9,10,24] make determination of their functional role difficult, without a clear lead regarding the specific vomeronasal pathways and chemosignals involved.

It is tempting to speculate that the post-mating increase in TH-positive periglomerular neurons in the AOB might be involved in preventing pre-implantation pregnancy block, via selective dopaminergic inhibition of vomeronasal input from mating male chemosignals. Future experiments using local infusions of dopaminergic antagonists would be required to test whether dopaminergic transmission in the AOB is indeed required for mate recognition, during the pre-implantation period. However, such a mechanism seems unlikely to be the sole determinant, as C57BL/6 females show normal mate recognition [23], despite low numbers of TH-positive neurons in the AOB and no significant post-mating increase in TH-expression. The low level of TH-expression in C57BL/6 females is likely to reflect a difference in overall dopaminergic phenotype, as low numbers of TH-positive neurons are observed in other brain areas of C57BL/6 mice [32]. This strain difference in TH-expression in the AOB may at least partly explain the failure of Serguera et al. [8] to observe TH-positive neurons in the AOB of the C57BL/6 females in their study. However, our observation of a small number of TH-positive neurons in the AOB of C57BL/6 females, using the same primary antibody, suggests that this discrepancy may partly also be explained by methodological differences. Notably, Serguera et al. (2008) post-fixed their tissue overnight, which we have found to reduce the sensitivity of immunohistochemical staining using this antibody. Furthermore, their procedure for counting neurons in every sixth coronal section may have missed the sparsely distributed neurons that were detected in our analysis of 16 consecutive sagittal AOB sections. Serguera et al.’s report of a dopaminergic gating of the MOB-mediated post-implantation pregnancy block is not inconsistent with the vomeronasally mediated pre-implantation block, which is the focus of this study. However, Serguera et al’s conclusions did depend on interpreting the effects of systemic injections of D_2_ antagonists assuming the lack of TH-positive neurons in the AOB [7,8]. Our findings of TH-positive neurons in the AOB suggest that the involvement of dopaminergic inhibition of MOB input in post-implantation pregnancy block should therefore be confirmed using more selective methodologies that completely eliminate the possibility of AOB-mediated effects.

In conclusion, our findings support the hypothesis that mate recognition in mice prevents pregnancy block by suppressing activation of arcuate dopaminergic neurons during exposure to chemosignals from the mating male. We found an increase following mating in the number of likely dopaminergic neurons at both the AOB input and the arcuate hypothalamic output of the pregnancy block pathway. Whether, this post-mating increase in dopaminergic transmission plays a role in the gating of the mate’s pregnancy blocking signal remains to be determined. Nevertheless, these findings highlight the possibility that specific vomeronasal inputs may be differentially modulated during the post-mating period, in female mice, and suggest new areas for future research.

## Supporting Information

Figure S1 Strain of male pre-exposure affects subsequent c-Fos expression in the AOB in response to male chemosignals. Mean (± standard error) number of c-Fos positive nuclei in glomerular and mitral cell layers of the anterior and posterior AOB of females that had been initially exposed/mated to either BALB/c or C57BL/6 male chemosignals. Single-factor ANOVA revealed a significant difference across groups for number of c-Fos nuclei in the anterior mitral cell layer of females that had been initially exposed/mated to BALB/c males. Post-hoc Dunnett’s comparison revealed significantly higher number of c-Fos positive nuclei in the exposed group than control group (*p = 0.017).
(TIF)
