# Supplementary figures and images for: Mating Increases Neuronal Tyrosine Hydroxylase Expression and Selectively Gates Transmission of Male Chemosensory Information in Female Mice
Source: PLoS One. 2013 Jul 25;8(7):e69943. doi: 10.1371/journal.pone.0069943 (PMC3723660; doi:10.1371/journal.pone.0069943)

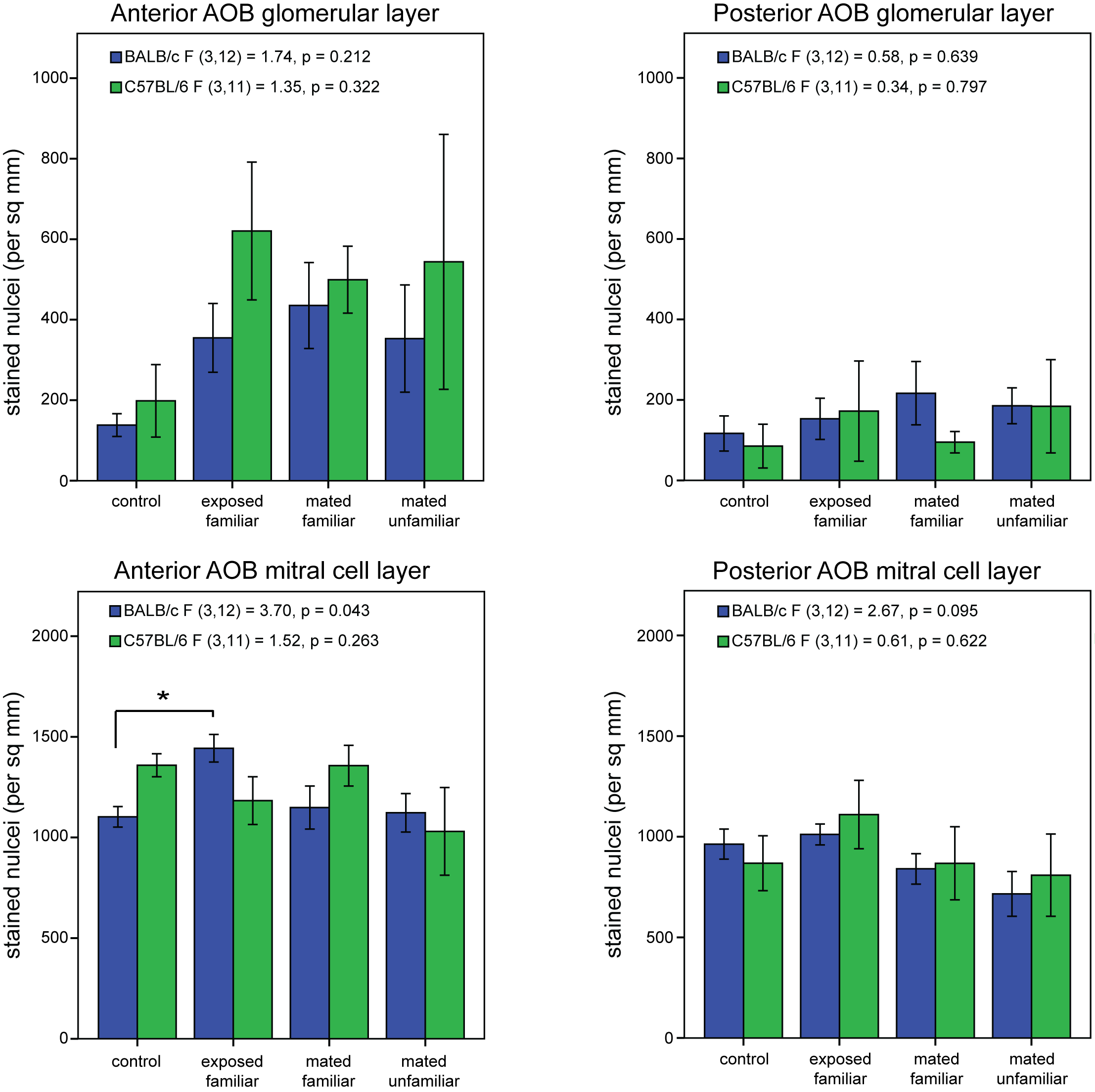

Supplement: Figure S1 — Mean (± standard error) number of c-Fos positive nuclei in glomerular and mitral cell layers of the anterior and posterior AOB of females that had been initially exposed/mated to either BALB/c or C57BL/6 male chemosignals. Single-factor ANOVA revealed a significant difference across groups for number of c-Fos nuclei in the anterior mitral cell layer of females that had been initially exposed/mated to BALB/c males. Post-hoc Dunnett’s comparison revealed significantly higher number of c-Fos positive nuclei in the exposed group than control group (*p = 0.017). (TIF) [file pone.0069943.s001.tif]
